# Blatchford Score Is Superior to AIMS65 Score in Predicting the Need for Clinical Interventions in Elderly Patients with Nonvariceal Upper Gastrointestinal Bleed

**DOI:** 10.1155/2016/6850754

**Published:** 2016-08-28

**Authors:** Khalid Abusaada, Fnu Asad-ur-Rahman, Vladimir Pech, Umair Majeed, Shengchuan Dai, Xiang Zhu, Sally A. Litherland

**Affiliations:** Florida Hospital Internal Medicine Program, Florida Hospital, 601 Rollins Street, Orlando, FL 32803, USA

## Abstract

*Background*. Blatchford and AIMS65 scores were developed to risk stratify patients with upper gastrointestinal bleed (UGIB). We sought to assess the performance of Blatchford and AIMS65 scores in predicting outcomes in elderly patients with nonvariceal UGIB.* Methods*. A retrospective cohort study of elderly patients (over 65 years of age) with nonvariceal UGIB admitted to a tertiary care center. Primary outcome was a combined outcome of in-hospital mortality, need for any therapeutic endoscopic, radiologic, or surgical intervention, rebleeding within 30 days, or blood transfusion. Secondary outcome was a combined outcome of in-hospital mortality or need for an intervention to control the bleed.* Results*. 164 patients were included. The primary outcome occurred in 119 (72.5%) patients. The secondary outcome occurred in 12 patients (7.2%). Blatchford score was superior to AIMS65 score in predicting the primary outcome (area under the receiver-operator curve (AUROC) 0.84 versus 0.68, resp., *p* < 0.001). Both scores performed poorly in predicting the secondary outcome (AUROC 0.56 versus 0.52, resp., *p* = 0.18).* Conclusions*. Blatchford score could be useful in predicting the need for hospital based interventions in elderly patients with nonvariceal UGIB. Blatchford and AIMS65 scores are poor predictors of the need for a therapeutic intervention to control bleeding.

## 1. Introduction

Upper gastrointestinal bleeding (UGIB) is a frequent presenting symptom in the elderly. The elderly, defined as individuals 65 years of age or older, form a population that has a high percentage of multiple comorbidities with related polypharmacy, including the concomitant use of antiplatelet treatments, which puts them at higher risk for gastrointestinal bleed and for clinical decompensation under the stress caused by acute bleeding [1].

The most common cause of nonvariceal UGIB in elderly patients is peptic ulcer disease and gastritis/esophagitis [2–6]. In-hospital mortality from UGIB in elderly patients in previous reports ranged between 0 and 8.4% [1–3, 7]. However, the studies that reported the higher mortality rates included variceal bleed [1, 3, 7].

Despite being a common presenting symptom, there is little information in the literature to guide physicians on the management of nonvariceal UGIB in the elderly.

Blatchford score was developed to predict a composite outcome of inpatient mortality, in-hospital rebleeding, endoscopic or surgical intervention, and need for blood transfusion in the general population presenting with UGIB [8]. Blatchford score was proposed as a tool to triage patients with UGIB to outpatient versus inpatient treatment [9–11].

Recently, the AIMS65 score was derived from a large database of UGIB patients to predict mortality [12]. Comparison of the two scores had conflicting results in the general population [13, 14]. In addition, the performance of Blatchford and AIMS65 scores in elderly patients has not been validated.

In this study we sought to compare the performance of Blatchford and AIMS65 scoring systems in predicting clinically meaningful outcomes and the need for an intervention to control the bleeding in elderly patients with nonvariceal upper GIB.

## 2. Methods

This is a retrospective cohort study. All research reviews were conducted under protocols approved by the local Institutional Review Board (IRBNet# 519910) and all data were collected and analyzed in a HIPAA compliant manner to ensure patient privacy and data integrity.

The study was conducted in a large tertiary care center. Patients over the age of 65 years admitted to our institution between 2009 and 2011 with a diagnosis of GIB from any source were identified using International Classification of Disease Ninth Edition (ICD9) codes. After the identification of patients with a diagnosis of GIB, charts were reviewed and data was extracted by trained internal medicine residents. All reviewers received training on data extraction and a database was created to standardize the process. Patients with known liver cirrhosis, presentation with acute liver failure, or history of variceal bleed were excluded. Patients also were excluded if GIB was not the presenting symptom to the emergency department (ED).

For each patient included in the study, the following data were collected through manual chart review: age, sex, medical history, albumin level, international normalized ratio, blood urea nitrogen, hemoglobin, systolic blood pressure on admission, pulse, changes in mental state including syncope and dizziness, presence of melena, associated comorbidities and medication use, findings on colonoscopy and esophagogastroduodenoscopy (EGD), other procedures, and pathology reports.

The scores of interest were Blatchford score and AIMS65 score. Each of these scores was calculated using information available at time of presentation to ED.

The Blatchford score was calculated from eight clinical or laboratory variables as defined by Blatchford et al. [8]. AIMS65 score was calculated from five clinical and laboratory variables as described by Saltzman et al. [12] (Table 1). Altered mental status in AIMS65 score was defined as a change in baseline mental or neurologic state documented by the ED physician or admitting physician at time of admission including dizziness, syncope, and presyncope.

Upper GIB bleed was defined as presentation with hematemesis or other UGIB symptoms with source of bleeding in the upper GI tract identified by endoscopy or other imaging studies such as a bleeding scan or angiography.

The primary outcome was a composite endpoint of inpatient mortality, readmission within 30 days for rebleed, need for blood transfusion, or need for any endoscopic, radiologic, or surgical intervention. The secondary outcome was a combined outcome of in-hospital mortality or need for an intervention to control the source of bleeding.

## 3. Statistical Analysis

Baseline characteristics and outcomes were summarized by frequency tabulation and means with standard deviations as appropriate. Discriminative ability of the scoring systems for predicting outcomes was evaluated by receiver-operator characteristic curve analysis.

The area under the receiver-operating characteristic curve (AUROC) was calculated and compared for both scores using the DeLong test [15]. A cutoff point was selected according to the maximal sum of the sensitivity and the specificity for each score. Patients were considered to be in the low risk group for each score if they fell below the cutoff point or in the high risk group if they were at or above the cutoff point. Estimates of sensitivity, specificity, and positive and negative predictive values were calculated for each score. Comparison between low and high risk groups for each score was performed using the chi score test and Fisher's exact test as appropriate. All statistical comparisons were 2 tailed, with *p* value < 0.05 considered statistically significant. The data analysis was performed by using STATA, version 13.0 (StataCorp, College Station, TX).

## 4. Results

One hundred and sixty-four (164) patients were included in the study based on inclusion and exclusion criteria. Demographic and clinical characteristics, treatments, and outcomes are shown in Table 2. No endoscopic investigations were done on 5% of the patients because either the patient declined the procedure or the treating physician decided against the procedure for clinical reasons.

In-hospital mortality was 0.63% (1 patient). This patient died from a sigmoid colon perforation after colonoscopy. Most of the patients (92.8%) in our cohort did not need a therapeutic endoscopic, surgical, or radiologic intervention. In these patients the bleeding stopped spontaneously with supportive and conservative management.

An intervention to control the bleeding source was performed in 9 patients (5.5%). Two patients (1.2%) needed a nonurgent intervention (Schatzki's ring dilatation and pancreatic cancer diagnosis with subsequent referral for palliative measures).

The composite outcome of in-hospital mortality, need for any intervention, readmission in 30 days for GIB, and need for blood transfusion occurred in 119 patients (72.5%); most of the outcome was driven by need for blood transfusion, which was required in 105 (64% of patients).

**Figure 1:**
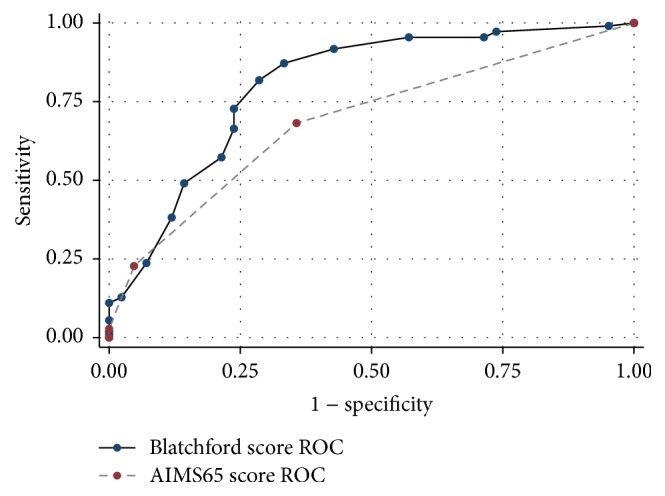
Area under receiver-operating characteristic (ROC) curve for Blatchford score and AIMS65 score in predicting composite outcome of in-hospital mortality, need for any intervention, readmission for rebleed within 30 days, or need for blood transfusion.

When comparing AUROC for predicting the primary composite outcome of in-hospital mortality, need for any intervention, readmission for rebleed within 30 days, or need for blood transfusion, Blatchford score (0.80 95% CI 0.71–0.88) was superior to AIMS65 score (0.68 95% CI 0.60–0.77) (*p* = 0.02) (Figure 1).

A Blatchford score more than or equal to 1 was able to identify 99% of high risk patients; however, only 3 patients (1.85% of the patients) had a score of 0. A Blatchford score more than or equal to 2 identified 97.5 of the high risk patients. Using a Blatchford score more than or equal to 2 as a cutoff resulted in 14 (8.5%) patients being classified as low risk. No patients in this group died or required an intervention; however, three patients received blood transfusion. This resulted in a sensitivity of 97.5% and specificity of 24.4% when a Blatchford score more than or equal to 2 is counted as high risk (Table 3). The primary outcome occurred in 77% of patients in the high risk group and in 21% in the low risk group (*p* < 0.001).

**Figure 2:**
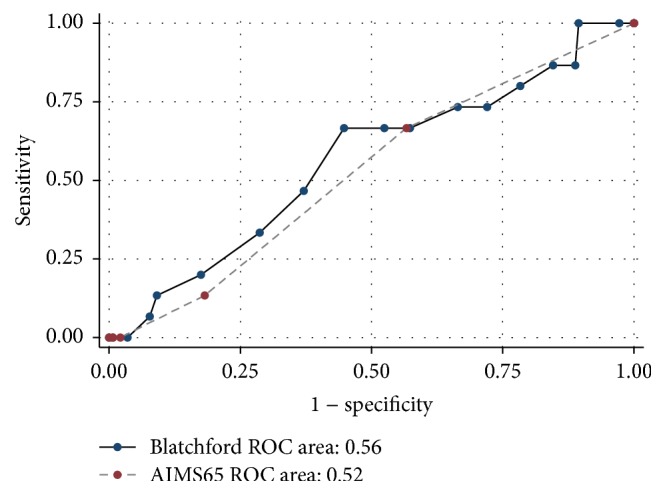
Area under receiver-operating characteristic (ROC) curve for Blatchford score and AIMS65 score in predicting the combined outcome of in-hospital mortality or need for urgent intervention to control bleeding.

Blatchford score and AIMS65 scores did not perform well in predicting the secondary outcome of in-hospital mortality or need for an intervention to control the bleeding [AUROC 0.56 (95% CI 0.34–0.70) versus 0.52 (95% CI 0.37–0.67)] for Blatchford and AIMS65 scores, respectively (*p* = 0.97) (Figure 2). Blatchford score more than or equal to 2 had 100% sensitivity and 100% negative predictive value for in-hospital mortality or need for an urgent intervention; however the specificity and the positive predictive value were very low (9 and 6.6%, resp.) (Table 4).

## 5. Discussion

The study evaluated the performance of Blatchford and AIMS65 scores in elderly patients presenting with nonvariceal upper GIB.

The low mortality rate in our cohort is within the range of mortality rates in elderly patients with nonvariceal upper GIB reported in prior studies [1–3, 7]. Prior studies on nonvariceal upper GIB in the general population had wide variation in mortality which is believed to be related to the definition of upper GIB and the populations studied [16]. We chose to define UGIB based on endoscopic findings rather than using presenting symptoms if other than hematemesis. In patients with GIB who do not present with hematemesis, an upper GI source is identified in only 30–74% of patients [17, 18]. In patients with hematochezia, it is estimated that about 10% have an upper source of bleeding identified [19, 20]. This makes the use of symptoms to define UGIB unreliable.

The need for an intervention to control bleeding in our cohort of elderly patient was also low (5.5%). This is much lower than rates of endoscopic interventions in prior studies of UGIB in the general population which ranged from 21 to 56% [21–25]. The low intervention and mortality rates in our study could be explained at least in part with the lower incidence of PUD in our population (27%) compared to other studies in which PUD represented 22–56% of the sample as well as exclusion of variceal bleeding. It has been suggested that the incidence of PUD in UGIB is exaggerated in studies of UGIB outcomes and it represents only about 20–30% of causes which is consistent with our findings [26]. This is likely related to differences in population and inclusion criteria used as some studies included variceal bleed [21–23] and patients who developed bleeding while hospitalized [22–24] and excluded patients who did not undergo endoscopy in the first 24 h of presentation [21, 24] and relied on clinical rather than endoscopic criteria to define upper GIB [21–23, 27]. For example, Pang et al. included only patients with UGIB who underwent an inpatient endoscopy within 24 hours in Hong Kong [21]. They had an intervention rate of 27% and a PUD incidence of 50%. 71% of the interventions were related to PUD and 15% were related to variceal bleed [21]. On the other hand, the multicenter study by Laursen et al. in Europe reported a PUD incidence of 31%, variceal bleed of 7%, and an intervention rate of 19% [27]. In addition, the lower rates of intervention could also be related to the older age of the population itself. Wang et al. reported an intervention rate of 9% in older adults (>60 years) with UGIB [25]. Our results are consistent with other studies in the elderly which reported that esophagitis [4] and gastritis [5] are the most common cause of nonvariceal GIB in older patients and are in line with other studies of the etiology of upper GIB in the elderly [6]. We believe that the population in our study is representative of the elderly population in the community.

Blatchford score was originally developed to predict similar outcomes in the general population presenting to the ED with an upper GIB. Stanley et al. [9] showed that outpatient management of patients with upper GIB with a Blatchford score of 0 is safe [9]. It has also been suggested that a threshold of Blatchford score more than or equal to 2 could be used as a decision cutoff for hospital admission in upper GIB [10, 11].

In our cohort of elderly patients only 1.8% of the patients had a Blatchford score of 0. This is largely due to increased prevalence of comorbidities and anemia in this population. A Blatchford score more than or equal to 2 identified 97.7 of the high risk patients. Blatchford score more than or equal to 2 has a high sensitivity for the combined outcome of in-hospital mortality, need for any intervention, and need for blood transfusion and readmission for rebleed in elderly patients with GIB. Even though the specificity of the test is very low using this cutoff, using Blatchford score more than or equal to 2 as a criterion for admission has the potential to reduce admissions for GIB by 8% in elderly patients. Although 3 patients (19%) in the low risk category required blood transfusion, none of them died or required an intervention to control the bleed and a Blatchford score more than or equal to 2 was able to identify 97.7% of the high risk patients. This high sensitivity and low specificity were pointed out by others [28] and are consistent with results of previous studies on the performance of Blatchford score in upper GIB in the general population [9–11].

AIMS65 score performed poorly in our cohort of elderly patients in predicting the need for admission. Our population was different from that whose AIMS65 score was originally derived and validated [12, 14]. We think that its reliance on age and albumin as factors in the score contributed to the poor performance in elderly patients with no advanced liver disease like ours.

Most elderly patients in our study stopped bleeding with supportive care only without the need for an intervention to control the bleed. However, both Blatchford and AIMS65 scores performed poorly in predicting the combined outcome of in-hospital mortality or the need for an intervention to control bleeding. A Blatchford score more than or equal to 2 has a very low specificity resulting in a low AUROC of 0.57 in predicting this outcome. Therefore, both Blatchford and AIMS65 scores have no clinical use in predicting which patients would benefit from an urgent intervention. However, Blatchford score more than or equal to 2 has 100% sensitivity for the secondary outcome. Therefore, a low score excludes the need for an intervention and a Blatchford score more than or equal to 2 can be used as a triaging tool to identify low risk patients who can be treated in the outpatient setting.

*Limitations*. The study is limited by the retrospective design of the study which may have compromised the data validity; however, the study was conducted using a structured protocol and training for data abstraction to limit bias. The study was conducted in a single institution which potentially could limit the generalizability of its results. In addition, 5% of the patients did not undergo endoscopic procedure; thus, the etiology of the bleed was uncertain; however, the outcomes of interest were available for those patients and exclusion of these patients could result in a selection bias. Furthermore, our results apply only to elderly patients with nonvariceal UGIB as patients with known liver disease or acute hepatic failure were excluded.

## 6. Conclusion

Nonvariceal UGIB in most elderly patients ceases spontaneously with supportive care without the need for an invasive intervention. Blatchford score is useful in predicting the need for hospital based interventions such as blood transfusion in elderly patient with nonvariceal UGIB and can serve as a triaging tool to identify low risk patients who can be treated as outpatients. However, it has low specificity and it should not be relied upon to predict the need for a therapeutic intervention. AIMS65 score is of no use to predict outcomes in elderly patients with UGIB. Further work is needed to develop better predictors of the need for an urgent intervention to control the bleed.

## Figures and Tables

**Table 1:** Blatchford score and AIMS65 score.

Risk factor	Score
Blatchford score	
*Blood urea (mmol/L) *	
≥6.5 <8.0	2
≥8.0 <10.0	3
≥10.0 ≤25.0	4
>25	6
*Hemoglobin (g/dL) for men *	
≥12.0 <13.0	1
≥10.0 <12.0	3
<10.0	6
*Hemoglobin (g/dL) for women*	
≥10.0 < 12.0	1
<10.0	6
*Systolic blood pressure (mm Hg) *	
100–109	1
90–99	2
<90	3
*Other factors*	
Pulse ≥ 100 (per min)	1
Melena	1
Syncope on presentation	2
Liver disease	2
Heart failure	2

AIMS65 score	
Albumin < 3.0 mg/dL	1
Age > 65	1
Altered mental status	1
Systolic blood pressure (mm Hg) < 90 mm Hg	1
INR > 1.5	1

INR, international normalized ratio.

**Table 2:** Patient characteristics and outcomes.

Characteristics	Number (%)
*Baseline characteristics*	
Age, mean (SD)	78.8 (0.54)
Gender (male)	75 (47%)
PPI at home	76 (46%)
NSAIDs at home	25 (15.2%)
Charlson comorbidity index, mean (SD)	5.79 (2.4)
*Presentation*	
Hematochezia	23 (14%)
Melena	95 (58%)
Hematemesis	53 (32%)
Unstable comorbidity on admission	13 (9.2%)
*Source of bleeding*	
Peptic ulcer disease	45 (27%)
Gastritis	75 (45%)
Duodenitis	8 (5%)
Esophagitis	44 (27%)
Neoplasm	1 (0.6%)
Normal endoscopy	5 (3%)
Mallory Weiss tear	1 (0.6%)
Arteriovenous malformation	7 (4%)
Ulcerated gastric polyp	3 (2%)
No endoscopy	9 (5%)
*Management and outcomes*	
Upper endoscopy only	100 (60%)
Colonoscopy only	2 (1.2%)
Upper endoscopy and colonoscopy	54 (33%)
No endoscopic procedure	8 (5%)
In-hospital mortality	1 (0.6%)
Intervention to control bleeding source	9 (5.5%)
Need for a nonurgent intervention to treat findings on endoscopic procedure	2 (1.2%)
Readmission in 30 days for rebleeding	33 (20.5%)
Received blood transfusion	105 (64%)

PPI: proton pump inhibitor, NSAIDs: nonsteroidal anti-inflammatory drugs, and SD: standard deviation.

**Table 3:** Performance of the scores in predicting the combined outcome of inpatient mortality, need for any intervention, readmission for rebleed within 30 days, and need for blood transfusion.

Score	Sensitivity %	Specificity %	PPV %	NPV %	AUROC (95% CI)
Blatchford score ≥2	97.5	25	79	81	0.80 (0.71–0.89)
AIMS65 score ≥2	68	64	83	43	0.68 (0.60–0.77)

PPV: positive predictive value. NPV: negative predictive value. AUROC: area under receiver-operator curve.

**Table 4:** Performance of the scores in predicting inpatient mortality or need for an urgent intervention to control bleeding.

Score	Sensitivity %	Specificity %	PPV %	NPV %	AUROC (95% CI)
Blatchford score ≥2	100	9	6.6	100	0.56 (0.34–0.70)
AIMS65 score ≥2	70	41.5	6	92.7	0.52 (0.36–0.67)

PPV: positive predictive value. NPV: negative predictive value. AUROC: area under receiver-operator curve.

## Additional Points

*Institutional Review Board Approval*. All research reviews were conducted under protocols approved by the local Institutional Review Board (IRB) and all data were collected and analyzed in accordance with the ethical standards as laid down in the 1964 Declaration of Helsinki and its later amendments or comparable ethical standards.
